# The 5,7-Dimethoxyflavone Suppresses Sarcopenia by Regulating Protein Turnover and Mitochondria Biogenesis-Related Pathways

**DOI:** 10.3390/nu12041079

**Published:** 2020-04-13

**Authors:** Changhee Kim, Jae-Kwan Hwang

**Affiliations:** Department of Biotechnology, College of Life Science and Biotechnology, Yonsei University, Seoul 03722, Korea; changgml@gmail.com

**Keywords:** 5,7-dimethoxyflavone, skeletal muscle, sarcopenia, aging

## Abstract

Sarcopenia is a muscle disease featured by the loss of muscle mass and dysfunction with advancing age. The 5,7-dimethoxyflavone (DMF), a major flavone found in *Kaempferia parviflora,* has biological activities, including anti-diabetes, anti-obesity, and anti-inflammation. However, its anti-sarcopenic effect remains to be elucidated. This current study investigated the inhibitory activity of DMF on sarcopenia. Eighteen-month-old mice were orally administered DMF at the dose of 25 mg·kg^−1^·day^−1^ or 50 mg·kg^−1^·day^−1^ for 8 weeks. DMF not only stimulated grip strength and exercise endurance but also increased muscle mass and volume. Besides, DMF stimulated the phosphatidylinositol 3-kinase-Akt pathway, consequently activating the mammalian target of rapamycin-eukaryotic initiation factor 4E-binding protein 1-70-kDa ribosomal protein S6 kinase pathway for protein synthesis. DMF reduced the mRNA expression of E3 ubiquitin ligase- and autophagy-lysosomal-related genes involved in proteolysis via the phosphorylation of Forkhead box O3. DMF upregulated peroxisome proliferator-activated receptor-gamma coactivator 1 alpha, nuclear respiratory factor 1, and mitochondrial transcription factor A along with the increase of relative mitochondrial DNA content. DMF alleviated inflammatory responses by reducing the tumor necrosis factor-alpha and interleukin-6 serum and mRNA levels. Collectively, DMF can be used as a natural agent to inhibit sarcopenia via improving protein turnover and mitochondria function.

## 1. Introduction 

The roles of skeletal muscle, which represents a dynamic tissue responsible for significant body functions, are involved not only in maintaining the body temperature and nutrient storage, especially in the regulation of proteins through a systemic metabolism, but also in converting chemical energy to a mechanical force or power for physical performances and social activities. Several external and internal stimuli, such as hormones, exercise, foods, drugs, and others, influence the activities, functions, and/or physiology of the skeletal muscle [1]. Three stimuli, including inactivity, metabolic or chronic diseases, and natural aging, are well-known negative factors to induce muscle atrophy featured by the loss of muscle fiber size and muscle mass as well as the dysfunction of the skeletal muscle [2]. Along with lowering responses to therapeutic agents including drugs, muscle atrophy not only decreases the mobility and the exercise capacity but also increases the risks of complications, falls, and mortality, consequently deteriorating the quality of the individual’s life and health [1,3].

Age-related muscle loss coupled with its dysfunction is called sarcopenia [4]. Since 2016, sarcopenia has been classified as a disease in accordance with the International Classification of Disease, Tenth Revision, Clinical Modification (ICD–10–CM) Code [5]. With an increasing age, the sarcopenia becomes regarded as a significant concern, which poses a substantial social and economic burden. In spite of great trials to pursue and develop agents and therapies, they remain unsatisfactory due to little convincing evidence and efficacy or accompanying side effects [6,7]. Currently, exercise training has been considered as a primary treatment for the sarcopenia or the muscle atrophy [6,7]. However, there is a limitation to suggest exercise training as an intervention for sarcopenia because elderly people and sarcopenia patients are likely to be bed-ridden due to illness or frailty [1,2]. Thus, the lack of interventions against sarcopenia emphasizes the need to develop effective and safe agents for elder people.

Focusing on the overall molecular mechanisms related to the protein turnover as well as the mitochondrial biogenesis helps to understand the physiology and dysfunction of the aged muscle. This gives opportunities for the development of new agents against sarcopenia, since both the protein turnover and the quality of mitochondria are closely associated with the function and mass of skeletal muscle [8,9]. First, muscle mass is critically determined by the protein turnover representing an elaborate balance between protein degradation and protein synthesis. As a representative signaling cascade to increase skeletal muscle mass, the phosphatidylinositol 3-kinase (PI3K)-Akt pathway improves the abnormal protein turnover in which the rate of protein breakdown exceeds the rate of protein synthesis [10,11,12]. The activation of the PI3K-Akt pathway enhances the process of translation through the phosphorylation of the mammalian target of rapamycin (mTOR) and suppresses the proteolysis-related system called the ubiquitin-proteasome via inhibition of the Forkhead Box O (FoxO) protein [10,11,12]. Second, mitochondria are a major organelle to generate chemical energy for muscle function [13]. FoxO not only accelerates the ubiquitin-proteasome system but also activates the autophagy-lysosomal system responsible for the degradation of misfolded proteins and organelles, particularly mitochondria [12]. Conversely, the peroxisome proliferator-activated receptor gamma coactivator 1 alpha (PGC–1α) is a major target protein to improve mitochondria function since it acts as an inducer of mitochondrial biogenesis, which is a process to increase the quality and quantity of mitochondria [14].

Physiologically, low but chronic inflammation is one of main factors to induce development of sarcopenia [15]. Interleukin (IL)-6 and tumor necrosis factor alpha (TNF–α), which represent major inflammatory cytokines, play majors role in accelerating the rate of protein degradation, deteriorating mitochondrial function, and excessively inducing inflammatory responses [16,17,18]. Once pro-inflammatory cytokines reach the membrane of muscle through blood circulation, they activate nuclear factor kappa B (NF–κB) pathway, subsequently increasing the expression of pro-inflammatory cytokines and inducing excessive inflammatory responses [19,20]. In terms of protein turnover related pathway, pro-inflammatory cytokines not only inactivate PI3K-Akt pathway [10,11] but also upregulate muscle ring-finger protein-1 (MuRF1), a major regulator of ubiquitin proteasome system for proteolysis [21]. They also damage organelles and cellular molecules, including DNA [17]. Not only damaging mitochondria, TNF-α blocks the process of mitochondrial biogenesis, giving rise to energy deprivation [18]. Thus, many anti-inflammatory agents including phytochemicals and flavonoids have been studied for their potential activities towards the prevention or slow-down of the progression of muscle atrophy [4,9,22].

**Figure 1:**
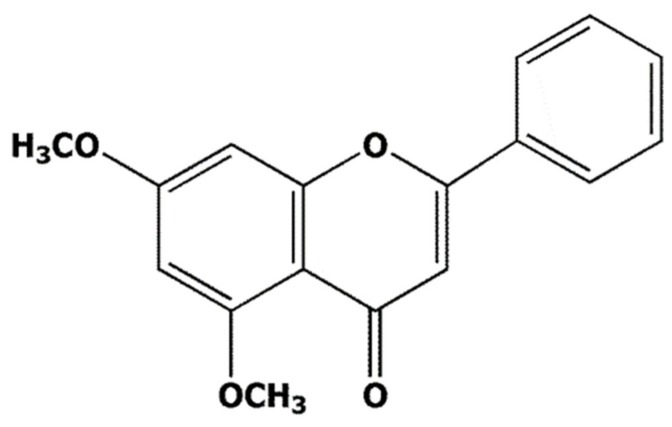
Chemical structure of 5,7-dimethoxyflavone (DMF).

The 5,7-dimethoxyflavone (DMF, Figure 1) is a major bioactive in *Kaempferia parviflora*, which has been used as food [23] and folk medicine to treat digestive disorders, gastric ulcer, and oral diseases [24]. DMF possesses a wide spectrum of pharmacological and biological activities, such as anti-obesity [25] and melanogenesis [26] functions. Previously, DMF has been shown not only to enhance energy metabolism by upregulating PGC-1α but also to increase glycogen contents and mRNA expression of glycogen synthase in the C2C12 myocytes [23,27]. In addition, the anti-inflammatory property of DMF has been well researched in various models [23,24,28]. These led us to the hypothesis that DMF potentially has anti-sarcopenic property. However, the anti-sarcopenic effect of DMF remains to be discovered. Here, we evaluated whether DMF delayed the development and progression of sarcopenia by focusing on the pathways related to protein turnover and mitochondrial biogenesis in the aged mice. Furthermore, we measured inflammatory cytokines in serum and muscle tissues to evaluate the role of an anti-inflammatory effect of DMF on the sarcopenic muscle.

## 2. Materials and Methods

### 2.1. Chemical Reagents

DMF was purchased from Aktin Chemicals, Inc. (Chengdu, China). Protease inhibitor cocktail and 2,2,2-tribromoethanol were supplied from Sigma-Aldrich (St. Louis, MO, USA). Primary antibodies against FoxO3 (Forkhead box O3), phospho (*p*)–FoxO3 (Thr32), mTOR, *p*–mTOR (Ser2448), 70-kDa ribosomal protein S6 kinase (*p*70S6K), *p*–*p*70S6K (Thr389), eukaryotic initiation factor 4E binding protein 1 (4EBP1), *p*–4EBP1 (Thr37/46), PI3K, *p*–PI3K (Tyr458), Akt, *p*–Akt (Ser473), and α-tubulin were purchased from Cell Signaling Technology (Beverly, MA, USA). Another primary antibody against nuclear factor kappa B (NF-κB) was from Santa Cruz Biotechnology Inc. (Santa Cruz, CA, USA). Horseradish peroxidase-conjugated goat anti-mouse and anti-rabbit antibodies were supplied from Bethyl Laboratories, Inc. (Montgomery, TX, USA). The NP-40 lysis buffer, 5× sample buffer, and Reverse Transcription (RT)-Premix were from Elpis Biotech (Daejeon, Korea).

### 2.2. Animal Experiments

Ten-week-old and 18-month-old C57BL/6J mice were purchased from the Laboratory Animal Resource Center (Korea Research Institute of Bioscience and Biotechnology, Cheongju, Korea). The mice were housed under a well-controlled environment (55 ± 5% humidity, 12-h day/12-h night cycles, and 25 ± 2 ℃) at the Yonsei Laboratory Animal Research Center (Seoul, Korea). Before performing the oral administration of DMF, the mice were acclimated to the experimental facility for 1 week. The 18-month-old mice were randomized into three groups as follows: (I) Aged, (II) Aged+DMF25, and (III) Aged+DMF50. The 10-week-old mice were designated to the young group (Young). The dosages were chosen according to previous study [25]. The mice in the Aged+DMF25 and the Aged+DMF50 groups were administered DMF at the dosages of 25 mg·kg^−1^·day^−1^ and 50 mg·kg^−1^·day^−1^, respectively, via oral gavage for 8 weeks, whereas the mice in the Aged and the Young groups received saline. During the oral administration, all mice had free access to food and water *ad libitum*. Body weight was measured once per week. Once oral administration was completed, the mice were anesthetized using 350 mg·kg^−1^ 2,2,2-tribromoethanol. From the anesthetized mice, blood samples were collected through a cardiac puncture. After the mice were sacrificed, four muscles from a hindlimb including the gastrocnemius (GA), the tibialis anterior (TA), the extensor digitorum longus (EDL), and the soleus (SOL) were carefully excised, weighed, and stored at −80 ℃. Before storage at −80 ℃, a small piece of GA muscle was fixed in 10% formalin (Junsei, Tokyo, Japan). All the experimental procedures were reviewed and approved by the Institutional Animal Care and Use Committee (IACUC) of Yonsei University (permit number: IACUC-A-201903-874-03).

### 2.3. Grip Strength Test

The grip strength of the mouse was assessed using the grip strength meter (Panlab, Barcelona, Spain). The forelimbs or the fore/hindlimbs of a mouse were placed on a grid, and then after visually confirming the gripping, the tail of mouse was gently pulled back until it released the grid. Five consecutive tests were performed per mouse and the average value was recorded as the grip strength of the mouse. 

### 2.4. Treadmill Test

Running time and running distance were evaluated using the treadmill (LE8710MTS, Panlab). Before starting the test, the electric shot at 0.2 mA was set, which was not harmful but was uncomfortable for the mice. Running test was initiated at 10 cm/s for 10 min on 0° incline, followed by increasing 1 cm/s per 1 min until the speed reached at 35 cm/s. The speed rate at 35 cm/s was maintained until the mice were exhausted. Exhaustion was defined as the inability to run for 10 s despite an electric shock. 

### 2.5. Histological Analysis

Fixed in 10% formalin, the GA muscle tissues were embedded in paraffin, cut into sections, and then mounted on slides. For histological analysis, the paraffin-embedded specimens were stained with hematoxylin and eosin (H&E). The random areas of the stained tissue were observed and captured using the CK40 inverted microscope (Olympus, Tokyo, Japan) equipped with a T500 camera (magnification, 200×; eXcope, Daejeon, Korea). The cross-sectional areas in the captured images were quantified with the Image J software (National Institutes of Health, Bethesda, MD, USA). Representative images of the quantified cross-sectional areas are represented.

### 2.6. Reverse Transcription-Polymerase Chain Reaction (RT-PCR) 

Total RNA from the GA and the SOL muscles were isolated using the Trizol reagent (Takara, Otsu, Japan). Purity and concentration of RNA were determined spectrophotometrically using the NanoDrop Lite spectrophotometer (Thermo Fisher Scientific Inc., Waltham, MA, USA). RNA was reverse-transcribed to cDNA with the RT premix at 42 ℃ for 60 min and then at 95 ℃ for 5 min using the SimpliAmp thermal cycler (Applied Biosystems, Foster City, CA, USA). PCR amplification was performed with the synthesized cDNA, the SafeDry Taq PCR premix (CellSafe, Gyeonggi, Korea), and specific primers (Bioneer, Daejeon, Korea) (Table 1). Amplification steps comprised 34–36 cycles as follows: Denaturation for 30 s at 95 ℃, annealing for 30 s at 58–59 ℃, and extension for 45 s at 72 ℃. The final step included extension for 5 min at 72 ℃. After that, the amplified products stained with the Loading star (DyneBio, Daejeon, Korea) were separated on 1.5% agarose gel and detected with the G:BOX EF imaging system (Syngene, Cambridge, UK) and the Gene Snap program (Syngene). The Image J software (National Institutes of Health) was used for densitometry analysis. The β-actin served as the internal control.

### 2.7. Western Blot Analysis

GA muscle tissues were homogenized with beads, and then lysed in the NP-40 lysis buffer containing 0.2% protease inhibitor cocktail on ice for 30 min. To remove insoluble materials, lysates were centrifuged at 16,000× *g* for 15 min, followed by collecting supernatants. Protein concentrations in the supernatants were determined using a Bradford solution (Bio-Rad, Hercules, CA, USA). Equal amounts of proteins were boiled with 5× sample buffer at 95 ℃ for 5 min, and then loaded and separated in 10–15% sodium dodecyl sulfate-polyacrylamide gel electrophoresis, followed by transferring onto a nitrocellulose membrane (GE Healthcare, Piscataway, NJ, USA). To block nonspecific binding, the membrane was exposed to 5% skim milk (Difco, Detroit, MI, USA) in Tris-buffered saline (Dynebio) containing Tween 20 (TBST). The membrane was incubated with primary antibodies for 18 h at 4 ℃, followed by washing three times for 10 min each with TBST. Next, the membrane was incubated again with secondary antibodies for 2 h at 4 ℃, and rinsed thrice with TBST for 10 min. Target proteins on the membrane were reacted with an enhanced chemiluminescence solution (Amersham Biosciences, Little Chalfont, UK) and then detected with the G:BOX EF imaging system (Syngene) and the GeneSys program (Syngene). The intensities of proteins were quantified by densitometry using the ImageJ software (National Institute of Health). The α-tubulin was served as the internal control.

### 2.8. Enzyme-Linked Immunosorbent Assay (ELISA)

Plasma was obtained by centrifuging blood samples at 1300× *g* for 15 min. TNF-α and IL-6 levels in plasma were quantified using a commercially available ELISA kit (R&D Systems, Minneapolis, MN, USA) following manufacturer’s instructions.

### 2.9. Analysis of Mitochondrial DNA Content

We performed the RT-PCR analysis with the SOL muscle according to the aforementioned method. The primer sequences of mitochondrial DNA (mtDNA) and genomic DNA (gDNA) (Bioneer) are shown in Table 1. The mtDNA/gDNA ratio was determined by measuring the relative band densities. 

### 2.10. Statistical Analysis

Statistical analysis was performed using SPSS 25.0 (SPSS Inc., Chicago, IL, USA). Significant differences between the Young and Aged groups were determined by the student’s *t*-test. Significant differences between the Aged and Aged+DMF groups were identified by one-way analysis of variance (ANOVA) followed by Duncan’s multiple-range test. Results with *p* < 0.05 were considered significant. All data were expressed as the mean ± standard deviation.

## 3. Results

### 3.1. DMF Enhances Physical Performances in Aged Mice

**Figure 2:**
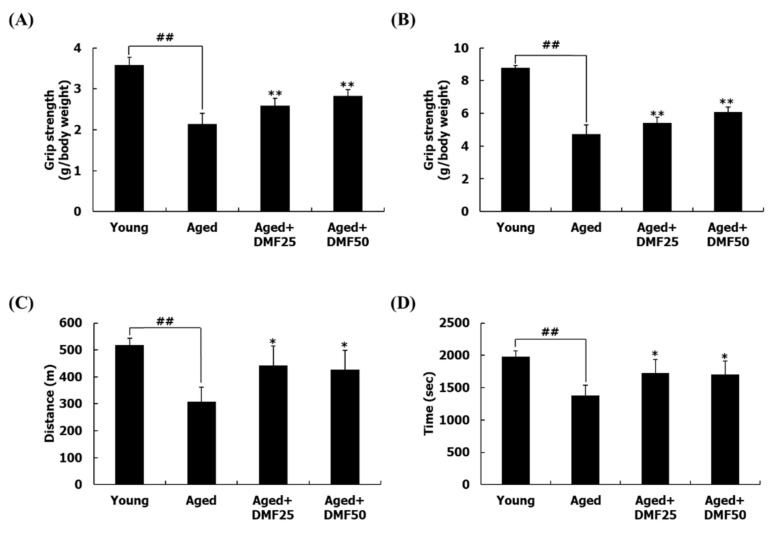
Effect of DMF on muscle function. (**A**) Forelimb strengths, (**B**) fore/hindlimb strengths. Grip strengths were normalized to the body weight. (**C**) Running distance, (**D**) running time. Results are presented as the mean ± SD; ^##^ *p* < 0.01 vs. Young group; * *p* < 0.05, ** *p* < 0.01 vs. Aged group.

From a mechanical perspective, poor physical performance is one of the major features of sarcopenia [8]. We evaluated the effect of DMF on physical performance in the aged mice by evaluating both the grip strength and the running endurance. The forelimb grip strength of mice in the Aged group was decreased by 40.29%, compared to that in the Young group; however, DMF treatments at the dosage of 25 mg·kg^−1^·day^−1^ and 50 mg·kg^−1^·day^−1^ dose-dependently increased this parameter by 20.91% and 31.84% in the Aged+DMF25 and the Aged+DMF50 groups, respectively (Figure 2A). In the case of the fore/hindlimb grip strength, the similar trend of the result of the forelimb grip strength was shown. The fore/hindlimb grip strength in the Aged group was 46.00% lower than that in the Young group, but the DMF treatment increased it by 14.23% and 28.52% in the Aged+DMF25 and Aged+DMF50 groups, respectively (Figure 2B). In comparison to the running time and distance in the Young group, those parameters in the Aged group were significantly decreased, while they were significantly elevated in response to the DMF administration. However, dose-dependent increases in running time and distance between the Aged+DMF25 and the Aged+DMF50 groups were not observed; the Aged+DMF25 group showed slightly higher parameters than those in the Aged+DMF50 group (Figure 2C,D).

### 3.2. DMF Increases Muscle Volume, Weights, and Cross-Sectional Area

**Figure 3:**
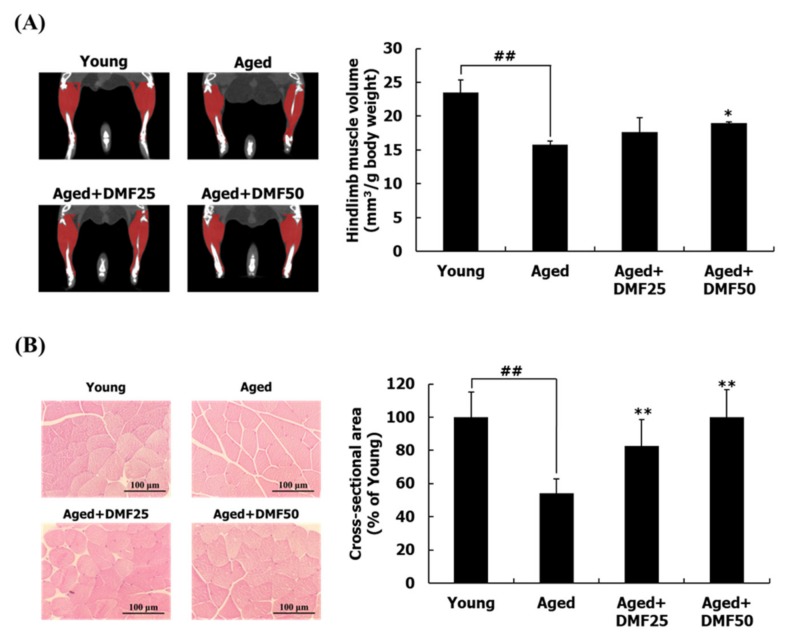
Effect of DMF on body composition. (**A**) Hindlimb volume. (**B**) The cross-sectional area of the gastrocnemius muscle fiber (magnification, ×200). Muscle volume was normalized to the body weight. Results are presented as mean ± SD; ^##^ *p* < 0.01 vs. Young group; * *p* < 0.05, ** *p* < 0.01 vs. Aged group.

Significant differences between the young mice and the aged mice were observed in both initial and final body weights (Table 2). Thus, we evaluated the muscle volume and the muscle weight by normalizing their values with body weights. The ratio of hindlimb muscle volume to body weight in the Aged group was significantly reduced to 67.21% of that in the Young group. However, DMF treatments at 25 mg·kg^−1^·day^−1^ and 50 mg·kg^−1^·day^−1^ increased the ratio of the hindlimb muscle volume/body weight, compared to that in the Aged group (Figure 3A). Aged mice had significantly lower GA, TA, EDL, and SOL muscle weights, compared to those in the young mice. DMF administration at the dosage of mg·kg^−1^·day^−1^ significantly increased all the muscle weights; however, in the Aged+DMF25 group, the GA, the EDL, and the SOL muscle weights were significantly increased except for that for the TA muscle, which was increased moderately but not significantly. All the muscle weights in the Aged+DMF groups were increased in a dose-dependent manner but did not reach similar levels as those in the young mice (Table 2). Consistently, the cross-sectional area of the GA muscle fibers, which were reduced by 45.83% in the Aged group in comparison to that in the Young group, was increased in a dose-dependent manner according to the DMF treatments (Figure 3B).

### 3.3. DMF Increases PI3K-Akt and mTOR Pathways

**Figure 4:**
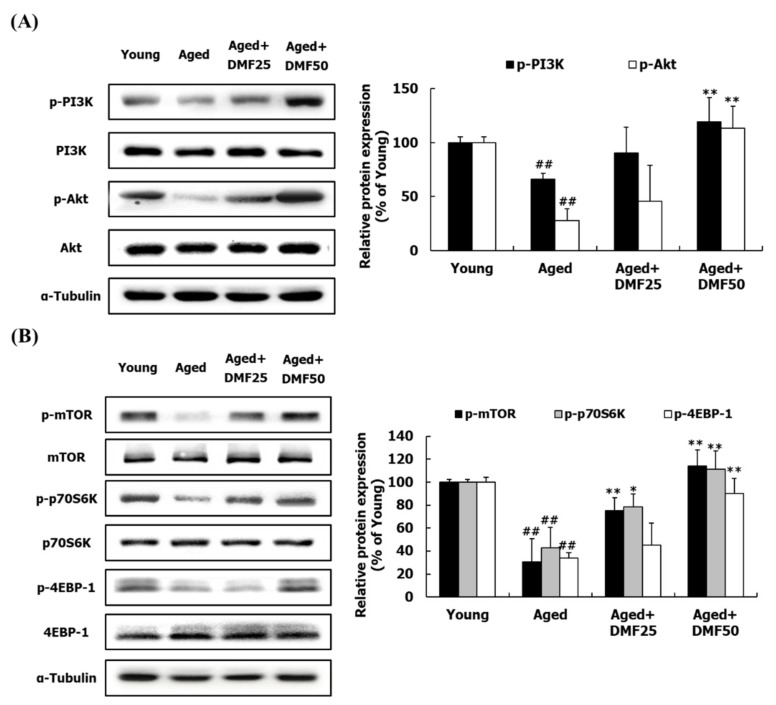
Effects of DMF on PI3K-Akt and mTOR pathways in the gastrocnemius muscle. (**A**) Relative phosphorylation of PI3K and Akt. (**B**) Relative phosphorylation of mTOR, p70S6K, and 4EBP1. Results are presented as mean ± SD; ^##^ *p* < 0.01 vs. Young group; * *p* < 0.05, ** *p* < 0.01 vs. Aged group.

Since the GA muscle occupies the most part of hindlimb muscle among the four muscles, we examined the molecular mechanisms related to the protein turnover by using the GA muscle tissue. The phosphorylation of PI3K and Akt in the Young group was higher than those in the Aged group. DMF treatment remarkably increased the phosphorylation levels of PI3K and Akt (Figure 4A). In addition to that, the reduced *p*-mTOR, *p*-p70S6K, and *p*-4EBP-1 in the Aged group were significantly recovered by DMF treatment. Particularly, DMF treatment led to an increase in the phosphorylation of mTOR and p70S6K, but not 4EBP-1 in a dose-dependent manner (Figure 4B). 

### 3.4. DMF Decreases FoxO Pathways

**Figure 5:**
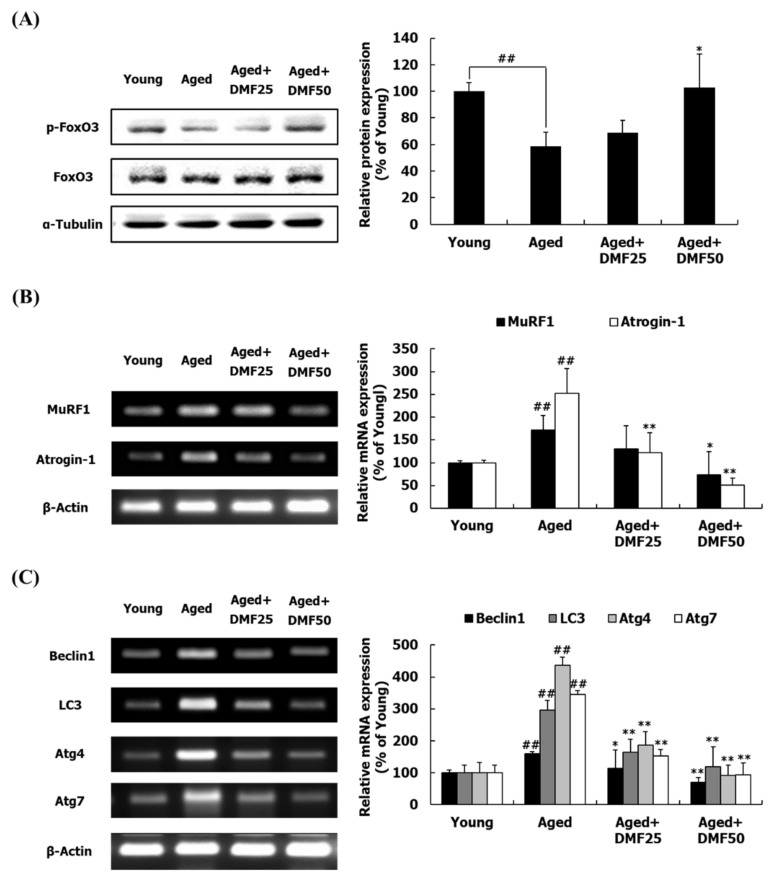
Effect of DMF on FoxO pathway in the gastrocnemius muscle. (**A**) Relative phosphorylation of FoxO. (**B**) Relative mRNA levels of MuRF1 and atrogin-1. (**C**) Relative mRNA levels of beclin1, LC3, Atg4, and Atg7. Results are presented as mean ± SD; ^##^ *p* < 0.01 vs. Young group; * *p* < 0.05, ** *p* < 0.01 vs. Aged group.

FoxO is a transcriptional factor that regulates the transcripts of genes which are related to the ubiquitin-proteasome and the autophagy-lysosomal systems. As an inactive form, p-FoxO cannot enter into the nucleus, thus remaining in the cytosol [11,12]. The phosphorylation of FoxO3, which was reduced in the Aged group, was upregulated in response to DMF treatment in the GA muscle (Figure 5A). This result suggests that DMF treatment prevents FoxO from translocating to the nucleus through phosphorylation. The mRNA expression of the MuRF1 and muscle atrophy F-box (MAFbx, another name is atrogin-1) was significantly upregulated by aging, while their expression levels were significantly reduced by DMF treatment (Figure 5B). The transcript patterns of biomarkers related to the autophagy-lysosomal system, including Beclin1, LC3, autophagy-related protein (Atg)4, and Atg7, was similar to the mRNA expression of MuRF1 and atrogin-1. As compared to the Young group, the mRNA expression levels of MuRF1 and atrogin-1 were significantly upregulated in the Aged group but downregulated in the Aged+DMF groups (Figure 5C).

### 3.5. DMF Increases Mitochondrial Biogenesis

**Figure 6:**
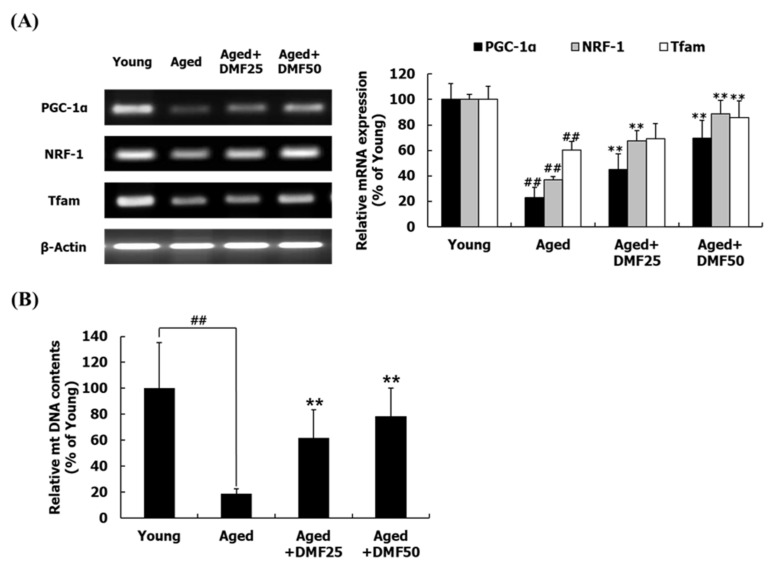
Effect of DMF on mitochondrial biogenesis in the soleus muscle. (**A**) Relative mRNA levels of PGC-1α, NRF-1, and Tfam. (**B**) Relative mtDNA contents. Results are presented as mean ± SD; ^##^ *p* < 0.01 vs. Young group; ** *p* < 0.01 vs. Aged group.

Comparison between type I, IIa, IIx, and IIb muscle fibers revealed that the mitochondria contents are the highest in the type I muscle fibers [13]. Among the four muscles consisting of the hindlimb, SOL muscle has been found to be abundant with type I fibers [29]; thus, in regard of mitochondrial biogenesis, we analyzed the gene expression using the SOL muscle tissue. In the Aged group, PGC-1α, nuclear respiratory factor 1 (NRF-1), and mitochondrial transcription factor A (Tfam) mRNA expression levels were markedly reduced as compared to those in the Young group. DMF treatment significantly increased the downregulated mRNA expression of PGC-1α, NRF-1, and Tfam; however, the expression levels of all markers did not reach similar levels as those in the Young group (Figure 6A). Consistently, relative mtDNA content was remarkably reduced in the Aged group, compared to those in the Young group. DMF administration, however, dose-dependently increased mtDNA content (Figure 6B).

### 3.6. DMF Attenuates Inflammatory Response

**Figure 7:**
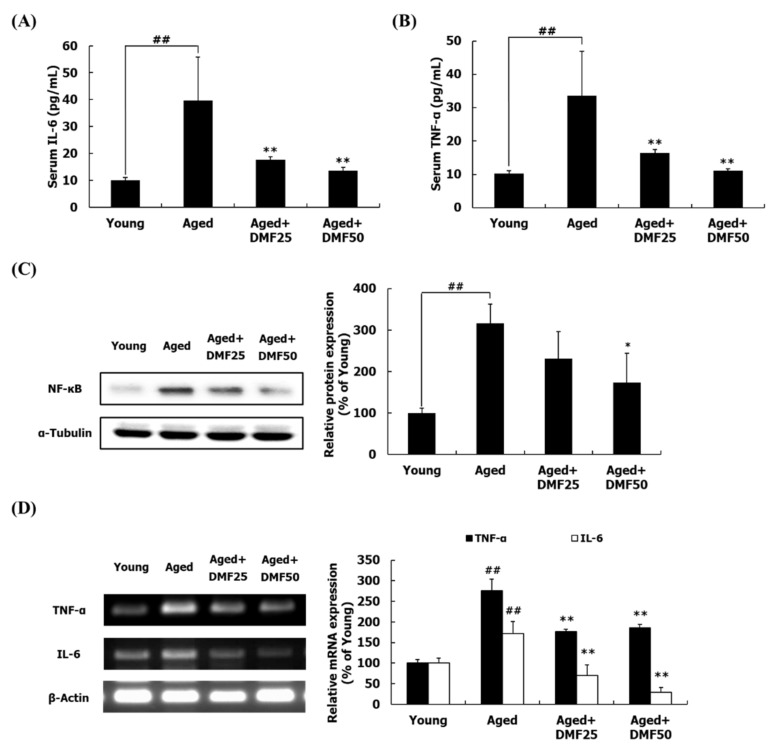
Effects of DMF on inflammatory responses in serum and the gastrocnemius muscle. (**A**) Serum IL-6 level. (**B**) Serum TNF-α level. (**C**) Relative protein level of NF-κB. (**D**) Relative mRNA levels of IL-6 and TNF-α. Results are presented as mean ± SD; ^##^ *p* < 0.01 vs. Young group; * *p* < 0.05, ** *p* < 0.01 vs. Aged group.

The effect of DMF on the inflammatory response was examined with serum and GA muscle tissue. In serum, the IL-6 and TNF-α levels were dramatically increased by 2.9- and by 2.3-fold, respectively, in the Aged group, as compared to those in the Young group (Figure 7A,B). However, DMF treatment significantly decreased the serum levels of IL-6 and TNF-α. When pro-inflammatory cytokines reach the membrane of muscle cells through the blood circulation, the inflammation through NF-κB is induced [15]. DMF treatment downregulated the protein expression level of NF-κB, which was upregulated in the Aged group (Figure 7C). Consistent with this result, the mRNA expression levels of IL-6 and TNF-α were elevated in the Aged group, but significantly decreased through the DMF treatment (Figure 7D).

## 4. Discussion

Sarcopenia represents an age-related muscle disease that has characteristics of loss of the muscle mass together with muscular dysfunctions including an abnormal metabolism and declines in the grip strength and physical performance [1,8]. Eighteen-month-old C57BL/6J mice are a well-established mouse model for studying natural aging, especially for sarcopenia [30]. In addition, according to the previous study, biomarkers related to old age were predominantly detected after at least 18 months [31]. Three-month-old mice are equivalent to 20-year-old humans and 18- to 24-month-old mice to 56- to 69-year-old humans [32]. Thus, 18-month-old C57BL/6J mice were employed to determine the inhibitory property of DMF on sarcopenia. In this study, we observed that grip strengths, exercise endurance, muscle volume, and muscle mass were significantly lower in the Aged group than those in the Young group, which indicates the occurrence of sarcopenia in this model. Conversely, the daily treatment with DMF for 2 months recovered all the reduced parameters (Figure 2 and Figure 3A–C). Thus, we suggest that DMF may have a potential activity against the progression and development of sarcopenia.

Next, we analyzed molecular mechanisms by which DMF prevents the development of sarcopenia. In developing novel agents to increase the muscle mass, it is essential to identify target molecules to improve the protein turnover. Our previous study showed that *K. parviflora* ethanol extract including 14.1% DMF inhibited obesity-induced muscle wasting by activating the PI3K-Akt pathway [33]. The PI3K-Akt pathway is of high interest to achieve this purpose [10,11], since it increases the protein turnover by simultaneous regulation of the protein anabolism and catabolism. The PI3K-Akt pathway regulates the downstream factors, mTOR, p70S6K, and 4EBP-1, leading to an increase in the protein synthesis [12]. In this study, DMF activated PI3K and Akt protein and increased the phosphorylation of mTOR, p70S6K, and 4EPB-1 (Figure 4). In addition to protein synthesis, FoxO3 acts as a key inducer of protein and mitochondria degradation through upregulation of genes related to the muscle-specific E3 ubiquitin ligases and the autophagy-lysosomal system; however, Akt prevents FoxO3 from being translocated into nucleus through phosphorylation [11,12]. Here, DMF increased FoxO3 protein phosphorylation and reduced the mRNA expression of MuRF1, atrogin-1, LC3, Becline1, Atg7, and Agt4 (Figure 5). In agreement with these results, icaritin and oligonol not only stimulated the mTOR pathway but also reduced the MuRF1 and atrogin-1 through activating the PI3K-Akt pathway, consequently increasing the muscle function and the muscle mass [8,34]. These results represent that DMF improved protein turnover in the aged muscle by stimulating the PI3K-Akt pathway. Overall, in the DMF-mediated process of the loss of muscle in the aged mice, the biomarkers involved in the PI3K-Akt signaling pathway and its downstream pathways can become the primary therapeutic targets. 

In addition to targeting PI3K-Akt pathway for protein turnover, mitochondria quality has been suggested as a new concept for the treatment of sarcopenia or muscle atrophy [8,9]. PGC-1α plays an important role in improving the mitochondrial function through mitochondrial biogenesis [14]. A complex with PGC-1α and NRF-1 works as a transcriptional factor of the Tfam mainly responsible for the mitochondrial DNA replication and transcription [14]. *K. parviflora* extract containing 14.1% DMF has increased PGC-1α, NRF-1, and Tfam mRNA expression as well as mitochondrial contents in vitro and in vivo [14,33]. DMF remarkably increased PGC-1α mRNA expression in C2C12 cells [27]. In agreement with these results, DMF upregulated the mRNA expression of PGC-1α, NRF-1, and Tfam in the aged SOL muscle, leading to an increase in the relative mtDNA contents (Figure 6). These results suggest that DMF stimulates mitochondrial biogenesis through the PGC-1α pathway in the aged mice; thus, DMF increases the mtDNA content through mitochondrial biogenesis. The β-adrenergic receptor (β-AR) agonist activates protein kinase A (PKA)–cyclic adenosine monophosphate (cAMP)-responsive element-binding protein (CREB) pathway, which improves both protein turnover pathway and mitochondrial biogenesis through PGC-1α isoform expression [35]. Previously, DMF treatment significantly increased cAMP in B16F10 cells in a dose-dependent manner [26]. It is conceivable that DMF might works as a β-adrenergic receptor agonist against sarcopenia; however, the relationship between DMF and β-adrenergic receptor will be needed as a further study.

Once pro-inflammatory cytokines reach the muscle through blood circulation, they reduce net protein contents and malfunctioning mitochondria, consequently inducing muscle atrophy [3,17,18]. Furthermore, pro-inflammatory cytokines upregulated TNF-α and IL-6, which act as autocrine and paracrine factors and then exacerbate the process of muscle wasting [20]. DMF has exhibited strong anti-inflammatory activity in several different models. For example, DMF reduced the production of inflammatory mediators, including IL-4 and TNF-α in the antigen-stimulated RBL-2H3 cells [28], and inhibited the release of TNF-α in the lipopolysaccharide (LPS)-activated RAW264.7 cells [24]. Furthermore, DMF decreased the mRNA expression of TNF-α and IL-6 in the LPS-treated C2C12 myotubes [23]. Here, DMF not only reduced the plasma levels of TNF-α and IL-6 but downregulated the NF-κB protein expression and TNF-α and IL-6 mRNA expression in the aged GA muscle (Figure 7). These results represent that decreases in serum TNF-α and IL-6 level following DMF treatment inactivate the NF-κB pathway, subsequently preventing the autocrine and paracrine effects of TNF-α and IL-6. It is conceivable that the reduced pro-inflammatory cytokines in response to DMF treatment improved PI3K-Akt pathway and stimulated mitochondrial biogenesis. Thus, the anti-inflammatory effect of DMF is involved in the prevention of sarcopenia. 

In this study, we discovered that the oral administration of DMF inhibited the development of sarcopenia in the aged mice. However, a previous study presented that DMF had no effect on the myotube diameter and the myosin heavy chain (MyHC) expression in the C2C12 myoblasts [36]. These discrepancies can be addressed via several explanations. First, based on the in vitro study, it is conceivable that DMF itself confers anti-atrophy rather than hypertrophic effect on the muscle of the aged mice. Second, this discrepancy is closely related to different experimental models. Some possibilities cannot be ruled out which can be only explained in animal model or clinical trials but not via an in vitro system. Bei et al. [37] provided scientific evidence that DMF was distributed to several tissues, such as the intestine, the liver, the brain, and fats, as well as the muscle through blood circulation after a single oral administration of DMF. Since the aging process seizes not just the muscle but also in all other tissues, DMF may not only directly improve the cell signaling in the sarcopenic muscle but also affect the aging process of other tissues, which indirectly but positively influence the muscle by a cross-talk with blood circulation. One of possible examples to support this hypothesis is the reduced plasma levels of IL-6 and TNF-α following DMF treatment (Figure 7A,B). Additionally, two metabolites of DMF including chrysin and 5-methoxy-7-hydroxyflavone, which were found after incubating DMF with human intestinal bacteria or orally administrating *K. parviflora* extract to rats [38,39], may influence the weight of the aged muscle. Further experiments will provide novel insights to prove these statements. 

## 5. Conclusions

This study investigated the inhibitory activity of DMF on sarcopenia. DMF treatment not only stimulated the grip strength and exercise endurance but also increased the muscle mass and volume in the aged mice. There are three points in DMF treating sarcopenia in the age mice. First, DMF stimulated the PI3K-Akt pathway, consequently activating the mTOR-p70S6K-4EBP1 pathway for protein synthesis and downregulating the mRNA expression of the E3 ubiquitin ligases- and the autophagy-related genes involved in proteolysis. Next, DMF stimulated the mitochondrial biogenesis by upregulating PGC-1α, NRF-1, and Tfam mRNA expression, as demonstrated by increase in the relative mtDNA content. Finally, DMF alleviated the inflammatory responses by reducing TNF-α and IL-6 levels in both serum and muscle. Collectively, DMF can be used as a natural agent to inhibit sarcopenia via improving the protein turnover and the mitochondrial function.

## Figures and Tables

**Table 1:** The sequences of forward and reverse primers for target genes.

Gene	Direction	Sequence (5’-3’)
MuRF1	Forward	TCTGGACTTAGAACACATAGCAGAG
	Reverse	TCTCCTTCTTCATTGGTGTTCTTCT
Atrogin-1	Forward	CAGTGATCCATTCTGTTCATCCTTG
	Reverse	TTATTTCCAGCCAAATGGAGAGAGA
Beclin1	Forward	CTCCTTAGGGGATGTTTGCCTT
	Reverse	TCAGGAAAGAGGGAAAGGATGC
LC3	Forward	GGGAGGTCCTGGCTCCTAAA
	Reverse	CAGACAGGCAAGGGCCTAAC
Atg4	Forward	GAGCCTTCCTCCATGTTCTTTCTC
	Reverse	CTCATATCTAGGGGGAGGAAAGGT
Atg7	Forward	TAGAGAGGACCTTGGCCTAACA
	Reverse	CAAGCCATCTATGTGTGTGCTG
PGC-1α	Forward	GAGTGTTCTGGTACCCAAGGC
	Reverse	CTGGGCCGTTTAGTCTTCCTT
NRF-1	Forward	AGATTCGTGGGTGGTAGGGT
	Reverse	TCTAGCAGAGGTCTAGGCGG
Tfam	Forward	AGACTACACTGGGAAACCACAG
	Reverse	GGCTTATAGGGACCCAGTGATG
IL-6	Forward	AGACAAAGCGAGAGTCCTTCAG
	Reverse	GTCCTTAGCCACTCCTTCTGTG
TNF-α	Forward	GAGTCATTGCTCTGTGAAGGGA
	Reverse	ATTCTGAGACAGAGGCAACCTG
Genomic DNA	Forward	GCCAGCCTCTCCTGATTTTAGTGT
	Reverse	GGGAACACAAAAGACCTCTTCTGG
Mitochondria DNA	Forward	CCGCAAGGGAAAGATGAAAGAC
	Reverse	TCGTTTGGTTTCGGGGTTTC
β-Actin	Forward	GAAGGAGATTACTGCTCTGGCTC
	Reverse	CTCAGTAACAGTCCGCCTAGAA

**Table 2:** Body and muscle tissue weights.

Parameter	Young	Aged	Aged+DMF25	Aged+DMF50
Body weight				
Initial weight (g)	21.92 ± 0.69	33.43 ± 0.86 ^##^	32.53 ± 0.78	32.90 ± 4.55
Final weight (g)	22.65 ± 0.78	35.12 ± 2.74 ^##^	35.26 ± 1.26	32.98 ± 5.57
Muscle weight /BW (mg·g^−1^)				
Gastrocnemius	10.56 ± 0.38	6.81 ± 0.77 ^##^	7.71 ± 0.45 *	8.01 ± 0.46 **
Tibialis anterior	4.30 ± 0.17	2.83 ± 0.13 ^##^	2.94 ± 0.23	3.12 ± 0.07 *
Extensor digitorum longus	1.87 ± 0.07	1.11 ± 0.08 ^##^	1.38 ± 0.13 **	1.48 ± 0.13 **
Soleus	0.74 ± 0.04	0.53 ± 0.04 ^##^	0.69 ± 0.07 **	0.71 ± 0.05 **

Results with *p* < 0.05 were considered significant; ^##^ *p* < 0.01 vs. Young group; * *p* < 0.05, ** *p* < 0.01 vs. Aged group.
